# Molecular Survey of Selected Tick-Borne Pathogens and Genetic Diversity of *Theileria equi* in Donkeys from Croatia

**DOI:** 10.3390/microorganisms14081840

**Published:** 2026-08-19

**Authors:** Nika Konstantinović, Daria Jurković Žilić, Antun Kostelić, Adnan Hodžić, Ana Marija Kovač, Gordan Šubara, Relja Beck

**Affiliations:** 1 Faculty of Veterinary Medicine, University of Zagreb, 10000 Zagreb, Croatia; nkonstantinovic@vef.unizg.hr; 2 Department for Bacteriology and Parasitology, Croatian Veterinary Institute, 10000 Zagreb, Croatia; jurkovic@veinst.hr; 3 Faculty of Agriculture, University of Zagreb, 10000 Zagreb, Croatia; akostelic@agr.hr; 4 Centre for Microbiology and Environmental Systems Science (CMESS), Department of Microbiology and Ecosystem Science, University of Vienna, 1010 Vienna, Austria; adnan.hodzic@univie.ac.at; 5 Department of Zootechnics, Križevci University of Applied Sciences, 48260 Križevci, Croatia; amkovac@vguk.hr; 6 Agency for Rural Development of Istria (AZRRI), 52000 Pazin, Croatia; gsubara@azrri.hr

**Keywords:** *Theileria equi*, donkey, Croatia, molecular epidemiology

## Abstract

Tick-borne pathogens remain insufficiently investigated in donkeys, particularly in southeastern Europe. This study investigated the occurrence of piroplasms, Anaplasmataceae, and hemotropic *Mycoplasma* spp. in donkeys from Croatia and assessed the genetic diversity of detected *Theileria equi*. Blood samples from 135 apparently healthy donkeys originating from 15 herds in six Croatian counties were examined using PCR. Positive *T. equi* samples were further characterized by sequencing, haplotype determination, and phylogenetic analysis. *Theileria equi* DNA was detected in 31/135 animals (22.96%; 95% CI: 15.99–29.94%), whereas no Anaplasmataceae or hemotropic *Mycoplasma* spp. DNA was detected. Infection showed marked geographical and herd-level variation. The highest prevalence was recorded in Šibensko-Kninska County (17/34; 50.0%), while all positive animals originated from three herds in the Croatian littoral region. Prevalence differed significantly among herd-size categories (*p* = 0.012), with no positive animals detected in small herds. Sequence analysis identified three *T. equi* haplotypes: D was predominant (22/31; 70.97%), followed by A (8/31; 25.81%) and E (1/31; 3.23%). Phylogenetic analysis confirmed all Croatian sequences as *T. equi* and indicated the presence of multiple genetic lineages within the sampled donkey population. These findings provide new molecular epidemiological data on *T. equi* in Croatian donkeys and demonstrate a heterogeneous distribution of infection among the investigated regions and herds. Among the selected tick-borne pathogens investigated, *T. equi* was the only pathogen detected. Further studies integrating larger and longitudinal datasets with detailed management, environmental, and tick-vector data are needed to better understand the factors underlying the observed epidemiological patterns.

## 1. Introduction

Tick-borne pathogens are of increasing importance in animal and public health due to their expanding distribution, driven by climate change, vector dynamics, and changing host–environment interactions [1]. Among tick-borne pathogens affecting equids, piroplasms *Theileria equi* and *Babesia caballi* are the causative agents of equine piroplasmosis (EP), a globally distributed disease affecting horses, donkeys, mules, and zebras and transmitted by ixodid ticks [2,3,4,5,6,7,8]. The disease is endemic in many parts of Southern and Central Europe [3], particularly in Mediterranean regions where environmental conditions favour the maintenance of competent tick vectors [4]. *Theileria equi* exhibits considerable genetic diversity, comprising five recognized 18S rRNA genotypes (A–E), while the closely related species *Theileria haneyi*, phylogenetically associated with genotype C, has recently been described [9]. Although the clinical manifestations of EP are well documented in horses, donkeys are more commonly reported as asymptomatic carriers capable of maintaining chronic infections and may contribute to the persistence of the disease by serving as reservoir hosts, particularly in endemic areas [10,11].

Acute infections may present with non-specific clinical signs, including fever, depression, anorexia, dehydration, eyelid oedema, constipation, icterus, and splenomegaly [12]. Following infection, equids infected with *Theileria equi* generally remain lifelong carriers, whereas *Babesia caballi* infections may be naturally cleared over time. These carrier animals contribute to parasite persistence and transmission in endemic regions [8]. Despite their recognized susceptibility, donkeys remain comparatively understudied with regard to the molecular epidemiology of piroplasm infections [2,4,8].

To date, molecular detection of *T. equi* in donkeys has been reported only sporadically in Europe. In Spain, the parasite was initially detected in a single donkey [5], while a recent study reported *T. equi* infection in one of two examined donkeys (50%) [13]. More extensive molecular data are available from Italy, where several studies have investigated equine piroplasms in donkeys. Veronesi et al. [10] detected *T. equi* DNA in 21 of 122 donkeys (17.2%), with genotypes A, B, and D identified among the positive animals, whereas *B. caballi* was detected in only one donkey. Similarly, Laus et al. [11] detected *T. equi* by PCR in 24 of 138 donkeys (17.4%), while *B. caballi* was detected in five animals (3.6%). In another Italian study, *T. equi* was detected in 36 of 109 donkeys (33.0%) [14]. In Serbia, *T. equi* infection was detected in 50% of apparently healthy donkeys, suggesting that the parasite is established in donkey populations in this part of southeastern Europe [15].

In addition to piroplasms, equids may be infected with other tick-borne pathogens, including *Anaplasma phagocytophilum*, the causative agent of equine granulocytic anaplasmosis, which has been increasingly reported throughout Europe [16]. In contrast, information on *A. phagocytophilum* infection in donkeys remains limited. DNA of *A. phagocytophilum* has been detected in 4% of apparently healthy donkeys in southern Italy [17], while Naranjo et al. [18] reported infection in all three examined donkeys from Spain. Data regarding hemotropic *Mycoplasma* spp. in donkeys are even scarcer, and their epidemiological significance remains poorly understood [19].

In Croatia, donkeys are predominantly kept under extensive or semi-extensive management systems in Mediterranean and sub-Mediterranean regions, where exposure to tick vectors is likely. However, apart from limited data on hemotropic *Mycoplasma* infections, no molecular investigations simultaneously targeting piroplasms, Anaplasmataceae, and hemotropic *Mycoplasma* spp. have been conducted in Croatian donkeys. Similarly, studies applying this multi-pathogen molecular approach remain scarce in Europe, with most published investigations focusing on a single pathogen group or a limited number of tick-borne agents. Therefore, the aim of this study was to investigate the occurrence of piroplasms, Anaplasmataceae, and hemotropic *Mycoplasma* spp. in donkeys from Croatia using molecular methods and to assess the genetic diversity of detected *T. equi* isolates through phylogenetic analyses.

## 2. Materials and Methods

### 2.1. Study Area, Animals and Study Design

The study was conducted in Croatia, which comprises Mediterranean, sub-Mediterranean, and continental climatic regions, with extensive karst landscapes in the coastal hinterland. Donkeys in Croatia are mainly distributed in coastal and sub-Mediterranean areas, particularly in Dalmatia and on the Adriatic islands, where they are commonly maintained under extensive or semi-extensive management conditions [20]. In these areas, animals are typically kept under extensive or semi-extensive management conditions, often grazing freely on natural pastures with minimal human intervention. In continental Croatia, donkeys are less numerous and are generally maintained in small private herds. Most donkeys were kept as breeding animals, whereas four individuals were used for recreational activities.

The total donkey population in Croatia is estimated at approximately 4000–5000 animals, most of which belong to three indigenous breeds such as the Istrian donkey, Littoral-Dinaric donkey, and North Adriatic donkey [20].

A total of 135 donkeys from 15 herds located in six Croatian counties were included in the study. Most animals originated from coastal and sub-Mediterranean regions: 47 donkeys were sampled in Istarska County, 34 in Šibensko-Kninska County, 29 in Dubrovačko-Neretvanska County, 10 in Primorsko-Goranska County, and nine in Ličko-Senjska County. An additional six donkeys were sampled in the continental part (Zagrebačka County). The investigated herds ranged from one to 34 sampled animals.

The investigated population comprised 10 males and 125 females. Available age records ranged from 1 to 19 years, with a mean age of approximately 4.5 years. Of the 135 examined donkeys, 131 were kept as breeding animals, whereas four were used for recreational activities. All animals underwent a clinical examination at the time of sampling and were considered apparently healthy, with no visible signs of clinical disease. Each animal was also inspected for the presence of attached ticks at the time of sampling; no ticks were detected on any of the examined donkeys.

The geographic locations of all sampled animals were recorded and mapped using QGIS (version 3.30.0), allowing visualization of the spatial distribution of the sampling sites across Croatia.

### 2.2. Sample Collection and DNA Extraction

Approximately 5 mL of EDTA-anticoagulated whole blood was collected from each animal by jugular venipuncture into sterile blood collection tubes (VACUETTE^®^, Greiner Bio-One, Kremsmünster, Austria). Samples were transported to the Laboratory of Parasitology, Croatian Veterinary Institute, within 48 h of collection, divided into aliquots, and stored at −20 °C until DNA extraction.

Extraction of genomic DNA was performed from 200 μL of anticoagulated blood using the automated KingFisher™ Flex platform (Thermo Fisher Scientific, Waltham, MA, USA) in conjunction with the MagMAX™ CORE Nucleic Acid Purification Kit (Thermo Fisher Scientific, Waltham, MA, USA), according to established high-throughput protocols.

### 2.3. Molecular Detection and Sequencing

Molecular screening was carried out using conventional PCR assays targeting three groups of vector-borne pathogens: piroplasms (*Babesia/Theileria* spp.), Anaplasmataceae, and hemotropic *Mycoplasma* species. Detection of Anaplasmataceae was based on amplification of a 345 bp fragment of the 16S rRNA gene using primers ehr16SR/ehr16SD [21]. *Babesia* and *Theileria* spp. were detected by amplification of an approximately 560 bp region of the 18S rRNA gene [22], while hemotropic *Mycoplasma* spp. were detected by targeting an approximately 600 bp fragment of the 16S rRNA gene [23]. After preliminary identification based on partial 18S rRNA gene sequences, all *Theileria equi*-positive samples were reanalysed using a PCR protocol targeting approximately 1700 bp of the 18S rRNA gene [24]. The resulting sequences were used for detailed haplotype determination and phylogenetic analyses.

PCR reactions were performed in a total volume of 20 μL containing 10 μL of GoTaq^®^ Green Master Mix (Promega, Madison, WI, USA), 0.5 μM of each primer, nuclease-free water, and 2 μL of template DNA. Amplification products were assessed using capillary electrophoresis on a QIAxcel instrument (Qiagen, Hilden, Germany).

Amplification products were evaluated by capillary electrophoresis using a QIAxcel system (Qiagen, Germany) with the QIAxcel DNA Fast Analysis kit (QIAGEN, Hilden, Germany), DNA QX Alignment Marker (15 bp–3 kb), and QX DNA Size Markers (50 bp–1.5 kb and 100 bp–2.5 kb). PCR products were purified using ExoSAP-IT™ PCR Product Clean-up Reagent (Applied Biosystems™, Thermo Fisher Scientific, Waltham, MA, USA) according to the manufacturer’s instructions and sequenced bidirectionally at Macrogen Europe (Amsterdam, The Netherlands) using the corresponding primer sets.

Raw sequence chromatograms were assembled and edited using SeqMan Pro version 18 and SeqBuilder Pro version 18 (DNASTAR, Madison, WI, USA), and consensus sequences were compared with reference data available in GenBank using BLAST (version 2.14.0, https://blast.ncbi.nlm.nih.gov, accessed on 20 June 2026) for species confirmation.

### 2.4. Statistical Analysis

The prevalence of *Theileria equi* infection was calculated as the proportion of PCR-positive animals among the total number of examined donkeys. Exact 95% confidence intervals (CI) for prevalence estimates were calculated using the Wilson method. In addition, a finite population correction (FPC) was applied to adjust the confidence intervals, based on an estimated total donkey population of approximately 4000 animals in Croatia.

Haplotype frequencies were determined based on sequence analysis and expressed as proportions of the total number of *T. equi*-positive samples. The distribution of haplotypes was calculated both within the positive subset and relative to the total sampled population.

Herds were categorized according to size as small (≤5 animals), medium (6–15 animals), and large (>15 animals). Associations between categorical variables (region and herd size) and infection status were evaluated using Fisher’s exact test. A *p*-value < 0.05 was considered statistically significant.

All statistical analyses were performed using Jamovi software (version 2.6.0; The Jamovi Project, Sydney, Australia).

### 2.5. Phylogenetic Analysis

The obtained *T. equi* 18S rRNA gene sequences were edited and aligned with representative reference sequences retrieved from GenBank^®^ using the ClustalW algorithm [25] implemented in BioEdit v7.2.5 [26]. Phylogenetic relationships were inferred using the neighbour-joining (NJ) method in MEGA 11 [27]. The most appropriate nucleotide substitution model was selected based on the corrected Akaike Information Criterion (AICc), with the Tamura-Nei model incorporating gamma-distributed rate variation among sites (TN93+G) providing the best fit to the dataset (AICc = 7986.743). The reliability of the inferred phylogenetic tree was assessed by bootstrap analysis with 1000 replicates.

## 3. Results

*Theileria equi* DNA was detected in 31 of 135 examined donkeys, corresponding to an overall prevalence of 22.96% (95% CI: 15.99–29.94%, finite population correction applied; estimated population size: 4000). No *Anaplasma/Ehrlichia* spp. or hemotropic *Mycoplasma* spp. DNA was detected in any of the examined samples.

The prevalence of infection varied markedly across the sampled regions. The highest prevalence was observed in Šibensko-Kninska County, where 17 of 34 animals tested positive (50.0%). In Istarska County, 12 of 47 donkeys were positive (25.5%), whereas a considerably lower prevalence was recorded in Dubrovačko-Neretvanska County (3/29; 10.3%). In contrast, no PCR-positive animals were detected among the sampled donkeys from Ličko-Senjska (0/9), Primorsko-Goranska (0/10), and Zagrebačka County (0/6). Statistical analysis showed that the prevalence of *T. equi* infection was significantly higher in Šibensko-Kninska County than in all other sampled counties (*p* = 0.0001). No statistically significant differences in prevalence were detected among the remaining counties (*p* > 0.05).

A total of 15 distinct herds were included in the study. Infection prevalence varied substantially between herds, with a clear contrast between completely negative and highly affected holdings. The highest prevalence was observed in the herd 12 (11/12; 91.7%), followed by herd 14 (17/34; 50.0%) while lower prevalence levels were recorded in herd 15 (3/29; 10.3%). Notably, no *T. equi* DNA was detected in the tested animals from more than half of the investigated herds (Table 1).

When analysed according to herd size, differences in *T. equi* prevalence were observed among the three herd-size categories. No positive animals were detected in small herds (≤5 animals; 0/16), whereas medium-sized herds (6–15 animals) showed the highest prevalence (11/33; 33.3%), followed by large herds (>15 animals; 21/85; 24.7%). The difference in prevalence among herd-size categories was statistically significant (*p* = 0.012), with small herds showing a significantly lower prevalence than medium- and large-sized herds.

**Figure 1:**
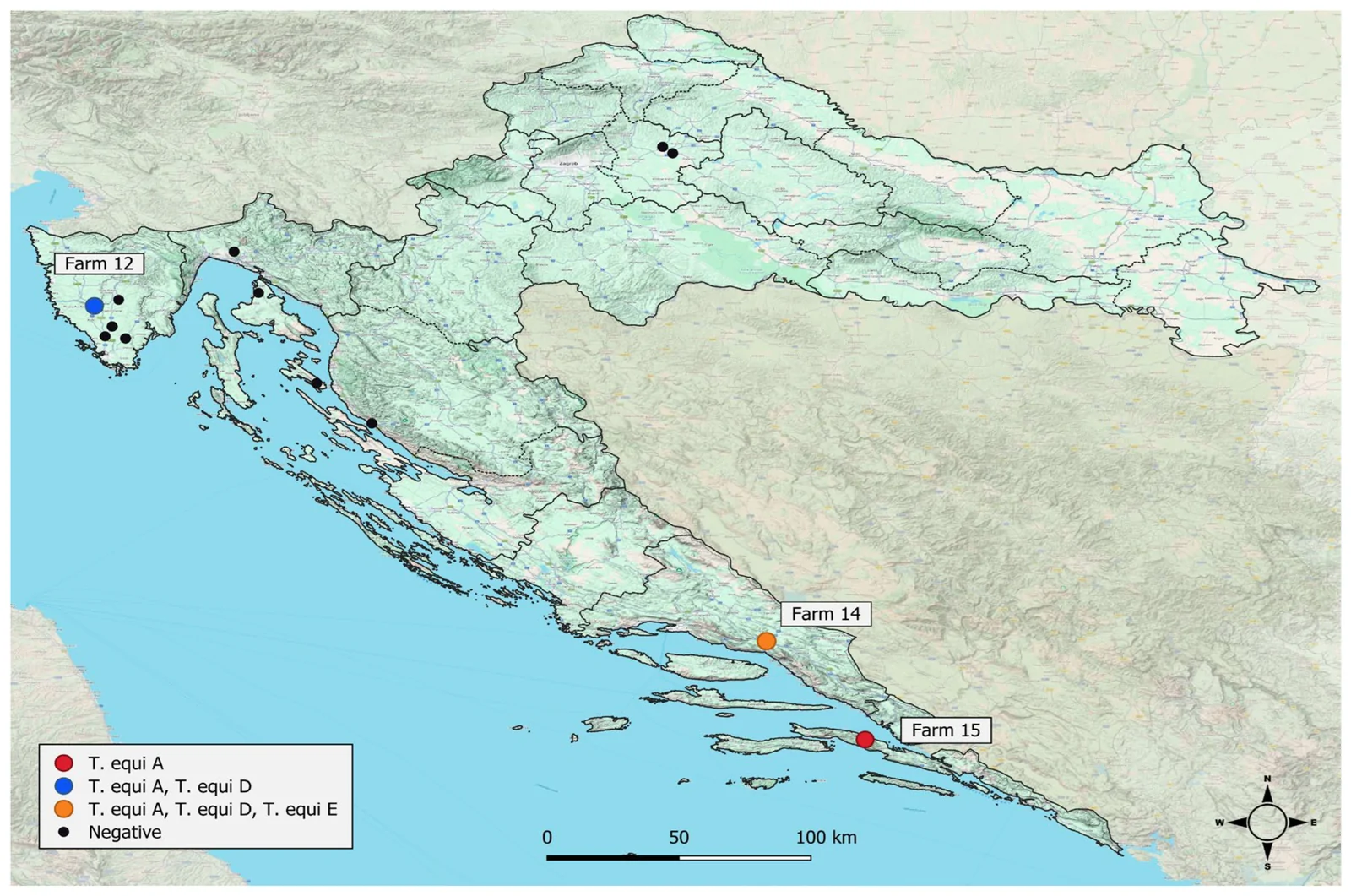
Spatial distribution of *Theileria equi* haplotypes in donkeys across Croatia. Sampling locations are shown with markers indicating haplotype composition: red—haplotype A; blue—haplotypes A and D; orange—haplotypes A, D, and E; black—PCR-negative animals.

Analysis based on sampling locations revealed a highly heterogeneous spatial distribution of *T. equi* infection. Positive cases were unevenly distributed and restricted to specific locations, while the majority of sampling sites remained negative (Figure 1). In the present study, all positive animals originated from three farms located in the Croatian littoral region, suggesting a geographically clustered distribution of *T. equi* infection within the sampled population.

Sequence analysis identified three distinct *T. equi* haplotypes. Haplotype D was the predominant variant, detected in 22 of the 31 positive samples (70.97%), followed by haplotype A, which was identified in 8 samples (25.81%). Haplotype E was detected in a single sample (3.23%).

When considered in relation to the total sampled population, haplotype D was present in 16.30% of animals, haplotype A in 5.93%, and haplotype E in 0.74%.

The spatial distribution of haplotypes was heterogeneous. The predominant haplotype (D) was distributed across multiple positive sampling locations, whereas haplotypes A and E showed a more restricted distribution (Figure 1).

**Figure 2:**
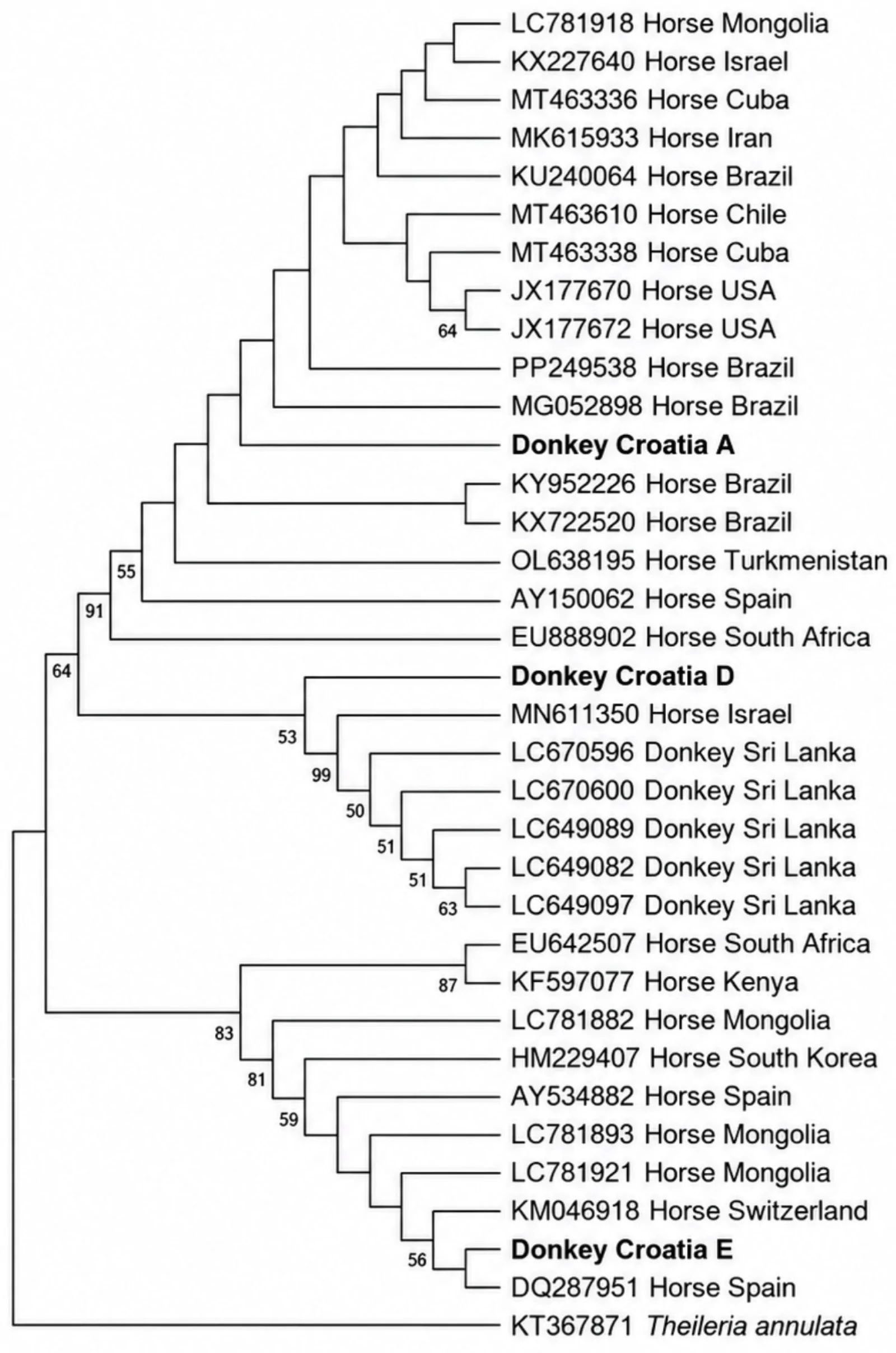
Phylogenetic tree of *Theileria equi* based on partial 18S rRNA gene sequences reconstructed using the Maximum Likelihood method. Croatian donkey isolates are indicated as Donkey Croatia A and Donkey Croatia E. Bootstrap values (>50%) are shown at branch nodes. *Theileria annulata* (KT387871) was used as an outgroup.

Phylogenetic analysis based on partial 18S rRNA gene sequences confirmed that all Croatian donkey isolates belonged to *T. equi*. The obtained sequences clustered within the main *T. equi* clade and were clearly separated from the outgroup species *Theileria annulata* (Figure 2). The nucleotide sequences generated in this study have been deposited in GenBank under accession numbers PZ596600–PZ596602.

The Croatian *T. equi* isolates were distributed among different phylogenetic subclusters together with sequences originating from Europe, Asia, Africa, and South America. Isolate Donkey Croatia A clustered with sequences from Brazil, South Africa, Spain, and other countries, whereas isolate Donkey Croatia E grouped with isolates originating from Switzerland, Mongolia, and Spain.

Phylogenetic analysis of the obtained sequences indicated that at least three genetic lineages, corresponding to haplotypes A, D, and E, are present in the sampled Croatian donkey population.

## 4. Discussion

The present study represents one of the few molecular surveys of selected tick-borne pathogens in donkeys from Croatia, addressing a host population for which molecular epidemiological data remain limited [4,8]. Epidemiological data on *Theileria equi* infection in donkeys from southeastern Europe remain limited [28]. Therefore, the present study provides additional information on the occurrence, distribution, and genetic diversity of this pathogen in this host species.

The results reveal a highly heterogeneous pattern of infection in Croatia, characterized by pronounced spatial variation, herd-level clustering, and clear differences associated with herd size. The overall prevalence of *T. equi* infection (22.96%) observed in the present study is comparable to values reported from Italy, where prevalences ranging from 17.4% to 33.0% have been described in donkeys [10,14]. However, it is considerably lower than the prevalence of 50% reported from neighbouring Serbia [15].

One of the most notable findings of the present study was the marked regional variability in *T. equi* infection. The significantly higher prevalence observed in Šibensko-Kninska County, together with the absence of positive animals in several other investigated regions, indicates a heterogeneous geographical distribution of infection. Positive animals originated exclusively from three herds located in the littoral region, whereas no *T. equi* DNA was detected in the examined animals from continental Croatia. Similar prevalence of *T. equi* infection in donkeys has also been reported from Mediterranean regions of Italy [10,11]. Such spatial heterogeneity may reflect differences in local ecological and environmental conditions, particularly those affecting the distribution and activity of competent tick vectors [2,4,6]. However, as climatic and environmental variables were not directly assessed in the present study, their contribution to the observed geographical pattern cannot be determined. Furthermore, the smaller number of donkeys sampled from continental Croatia should also be considered when interpreting the observed geographical differences.

Several species of *Hyalomma* and *Rhipicephalus* ticks have been reported from Croatia, including its Mediterranean and coastal regions [29,30]. Their known geographic distribution may therefore be relevant when considering possible ecological explanations for the observed pattern of *T. equi* infection. Supporting the biological plausibility of this hypothesis, a recent study from Sardinia detected *T. equi* and *Babesia caballi* DNA in ticks collected from free-ranging donkeys, including *Hyalomma marginatum* and *Rhipicephalus bursa* [31]. However, as ticks were not collected in the present study and their abundance, distribution, or infection status at the investigated localities was not assessed, any relationship between local tick populations and the observed geographical distribution of *T. equi* remains hypothetical and requires further investigation.

At the herd level, infection was clearly aggregated, with several herds showing high prevalence while more than half of the investigated herds were entirely negative. Herd size was significantly associated with *T. equi* infection, with a lower prevalence observed in small herds than in medium-sized and large herds. Previous studies have identified tick exposure and several host-, management-, and environment-related variables as potential risk factors for equine piroplasmosis, although associations with herd management and environmental factors have not been consistent among studies [2,10]. In the present study, detailed management practices, animal density, grazing conditions, and tick exposure were not systematically assessed. Therefore, although such factors may contribute to the observed association with herd size, the underlying mechanisms cannot be determined from our data and require further investigation.

Despite the detection of *T. equi*, all examined donkeys were clinically healthy at the time of sampling. This finding is consistent with previous studies reporting that donkeys frequently remain asymptomatic despite persistent *T. equi* infection and may maintain long-term parasitaemia without developing overt clinical signs [6,10,11].

Such chronically infected animals may serve as reservoir hosts, providing a potential source of infection for competent tick vectors [6]. The high prevalence observed in several herds in the present study is consistent with the hypothesis that donkeys may contribute to local transmission cycles of equine piroplasmosis, although this role requires confirmation through integrated epidemiological and vector studies.

An additional important finding of this study is the genetic diversity of *T. equi* circulating in Croatian donkeys. Sequence analysis identified three haplotypes, with haplotype D being clearly predominant (70.97%), followed by haplotype A (25.81%), whereas haplotype E was detected in only one animal (3.23%). A similar pattern has been reported in Italian donkeys, where Veronesi et al. [10] identified genotypes A, B, and D, confirming the predominance of haplotypes A and D in both countries. However, unlike the Italian study, in which genotype B was detected, the present study identified haplotype E, which was absent from the Italian donkey population. Interestingly, previous investigations of equine piroplasmosis in Croatia primarily reported haplotypes A and E in horses, whereas haplotype D was absent or detected only sporadically [32]. The predominance of haplotype D in the donkeys examined in the present study may indicate differences in the distribution of *T. equi* haplotypes between the previously investigated horse populations and the donkey population examined here. However, direct comparisons of horses and donkeys from the same geographical areas, together with analysis of the corresponding tick populations, would be required to determine whether this pattern reflects a true host-associated difference.

Phylogenetic analysis confirmed that all Croatian sequences belonged to *T. equi* and clustered with isolates from Europe, Asia, Africa, and South America. Although no clear geographic clustering was observed, Croatian isolates were distributed among several phylogenetic subclusters suggesting the presence of multiple genetic lineages within the analysed donkey population. Because the analysed 18S rRNA marker is relatively conserved, future studies using more variable genetic markers may provide greater resolution of the population structure of *T. equi* in Croatia.

An important additional finding is the absence of Anaplasmataceae and hemotropic *Mycoplasma* spp. in all examined animals. Despite its broad host range and ability to infect a wide variety of domestic and wild mammals [33,34], *Anaplasma phagocytophilum* appears to be rarely detected in donkeys. To date, molecular evidence of infection in European donkeys has been reported only from Sicily, Italy, where *A. phagocytophilum* DNA was detected in 5.2% (4/76) of examined animals [17]. Nevertheless, *Anaplasma phagocytophilum* has previously been detected in Croatia, including in the coastal region [35], indicating that the pathogen is present in at least some parts of this area. Moreover, Jaarsma et al. [34], in a large-scale study that also included samples from Croatia, demonstrated the occurrence of *A. phagocytophilum* in a broad range of domestic and wild vertebrate hosts, including horse. Therefore, the absence of Anaplasmataceae DNA in the examined donkeys may reflect a low prevalence of infection, limited exposure to infected tick vectors, or other epidemiological factors. Further studies including larger donkey populations and concurrent investigation of tick vectors would be needed to clarify the role of donkeys in the epidemiology of *A. phagocytophilum* in Croatia. In a previous Croatian study, hemotropic *Mycoplasma* spp. were not detected in donkeys [19] although the number of examined animals was considerably lower. Considering the relatively large sample size and broad geographical coverage of the present study, this finding indicates that hemotropic *Mycoplasma* infections were not detectable in the investigated Croatian donkey population. Whether this reflects a very low prevalence, limited exposure to potential sources of infection, or lower host susceptibility cannot be determined from the present data. Further studies would be required to distinguish between these possible explanations.

Several limitations of the present study should be considered when interpreting these findings. Although the investigated donkeys originated from six Croatian counties and different geographical regions, the number of sampled animals was unevenly distributed, with substantially fewer animals examined from continental Croatia. In addition, the cross-sectional design provides information on infection status only at the time of sampling and does not allow assessment of temporal changes in infection within individual animals or herds. Detailed information on management practices, grazing conditions, animal movements, contact with other equids, and previous exposure to ticks was not systematically available for all investigated animals. Furthermore, although all donkeys were inspected for attached ticks at sampling and none were detected, systematic tick collection was not part of the study design. Consequently, the presence, abundance, and infection status of potential tick vectors at the investigated locations could not be directly related to the observed distribution of *T. equi*. Future studies combining longitudinal sampling of donkeys with concurrent collection and molecular examination of ticks would therefore provide a more complete understanding of local transmission patterns. Inclusion of detailed management and environmental data, together with molecular characterization using additional and more variable genetic markers, could also help clarify the epidemiological factors associated with infection and the population structure of *T. equi* in Croatian equids.

## 5. Conclusions

This study represents one of the few molecular surveys of selected tick-borne pathogens in donkeys in Croatia and demonstrates a heterogeneous distribution of *T. equi* infection, with marked differences among geographical regions and herds. Three *18S rRNA* haplotypes were identified, with haplotype D clearly predominating, indicating genetic variation within the analysed *T. equi* population. No DNA of Anaplasmataceae or hemotropic *Mycoplasma* spp. was detected in the examined donkeys. Thus, among the pathogens investigated in the present study, *T. equi* was the only tick-borne pathogen detected. The observed geographical and herd-level patterns highlight the need for further studies integrating larger and longitudinal datasets with detailed management, environmental, and tick-vector data to better understand the epidemiology and transmission dynamics of *T. equi* in Croatian donkeys.

## Figures and Tables

**Table 1:** Distribution of the examined donkey farms, the number of sampled animals, number and prevalence of *Theileria equi*-positive animals, and haplotypes identified in positive farm.

Farm	County	No. of Animals	No. Positive	Prevalence	Haplotype(s)
Farm1	Primorsko Goranska	1	0	0	
Farm2	Primorsko Goranska	3	0	0	
Farm3	Istarska	7	0	0	
Farm4	Primorsko Goranska	6	0	0	
Farm5	Zagrebačka	3	0	0	
Farm6	Zagrebačka	3	0	0	
Farm7	Ličko Senjska	8	0	0	
Farm8	Ličko Senjska	1	0	0	
Farm9	Istarska	1	0	0	
Farm10	Istarska	1	0	0	
Farm11	Istarska	22	0	0	
Farm12	Istarska	12	11	91.7%	A, D
Farm13	Istarska	4	0	0	
Farm14	Šibensko Kninska	34	17	50.0%	A, D, E
Farm15	Dubrovačko Neretvanska	29	3	10.3%	A

## Data Availability

The original contributions presented in this study are included in the article. Further inquiries can be directed to the corresponding author.
